# Mechanical properties of highly porous PEEK bionanocomposites incorporated with carbon and hydroxyapatite nanoparticles for scaffold applications

**DOI:** 10.1007/s40204-019-00123-1

**Published:** 2019-10-19

**Authors:** Md. Nizam Uddin, Puttagounder S. Dhanasekaran, Ramazan Asmatulu

**Affiliations:** Department of Mechanical Engineering, Wichita State University, 1845 Fairmount St, Wichita, KS 67260-0133 USA

**Keywords:** Bone regeneration, Hydroxyapatite, Melt casting, Salt leaching, PEEK, Porous scaffolds

## Abstract

Bone regeneration is of great importance worldwide, because of various bone diseases, such as infections, tumors, and resultant fracture, birth defects, and bone loss due to trauma, explosion, or accident. Bone regeneration can be achieved by several materials and templates manufactured through various fabrication techniques. Uses of different materials and scaffold fabrication techniques have been explored over the past 20 years. In this research, polyetheretherketone (PEEK) was used to fabricate highly porous bionanocomposite foams for bone scaffolding. Melt casting and salt porogen (200–500 µm size) leaching methods were adapted to create an adequate pore size and the necessary percent of porosity, because pore size plays a vital role in cell implantation and growth. Porosity (75% and 85%) of the prepared scaffolds was adjusted by changing salt concentrations in the PEEK powder. Hydroxyapatite (HA) and carbon particles were used to improve cell attachments and interactions with the porous PEEK and to increase the mechanical properties of the scaffold materials. Carbon fiber (CF) and carbon nanotubes (CNTs) were uniformly dispersed into the PEEK powder before melt casting to enhance the mechanical properties and to observe the influence of the carbon particles on the properties of PEEK bionanocomposite foam. Compression test results of the fabricated bionanocomposites showed that HA and carbon particles are the potential filler materials for the enhancement of bionanocomposite mechanical properties. About 186% enhancement of compression modulus and 43% enhancement of yield strength were observed while incorporating only 0.5 wt% of CNTs into PEEK/HA bionanocomposites having 75% porosity, compared to PEEK/HA 20 wt% bionanocomposites. Micro-computed tomography (micro-CT) test results reveal that pore size and interconnectivity of the nanocomposite foams are in order and within the designed sizes. Mechanical tests proved that PEEK bionanocomposite foam has the potential for use in bone scaffolding and other biomedical applications.

## Introduction

The necessity for bone replacement has been drastically increasing due to accidents, disease, birth defects, military practices, and bone loss at a later age. The option of autografting or transplanting has risks including potential disease transmission and immunological rejection (Oryan et al. 2014; Polo-Corrales et al. 2014). As such, many researchers have extended their investigations for developing bionanocomposites for scaffold applications. The characteristics of ideal scaffolds for bone tissue engineering include the following: (1) interconnecting pores, including both macropores (pore size > 100 μm) and micropores(pore size < 20 μm) for tissue growth, substance transplantation, and vascularization; (2) biodegradable or bioabsorbable materials with strong mechanical properties to transfer load to the surrounding tissue; and (3) a good scaffold interface to adhere, proliferate, and differentiate cells efficiently (Saiz et al. 2013; Bose et al. 2012). Various materials, including biodegradable polymers such as polycaprolactone (PCL), polylactide (PLA), polyorthoester (POE), polyglycolide or polyglycolic acid (PGA), and their copolymers poly (lactic-*co*-glycolide) (PLGA) have been used to fabricate scaffolds, which could provide initial support and mechanical strength with adequate porosity for cell attachment (Seal et al. 2011; Mano et al. 2014; Seraz et al. 2017; Asmatulu et al. 2019). Thus, in the past few years, the combination of polymer/ceramic composites has gained much interest in the field of tissue engineering (Zanello et al. 2016; Abedin et al. 2015; Uddin et al. 2019a, b). The search for new bone-regeneration strategies is a key priority as the result of debilitating pain associated with bone damage and increasing medical and socioeconomic challenges of the aging population. It is extremely challenging to duplicate or reproduce organics and minerals found in vivo. The mechanical properties of current nanocomposites or composites are much lower than those of natural bone. However, polyetheretherketone (PEEK) composites have been gaining research interest as a load-bearing material in orthopedic applications because of its superior mechanical properties, biocompatibility, radiolucent and sterilization resistance, ease of processing, excellent wear, and fatigue properties, high-temperature performance, chemically inertness, and lack of toxicity (Williams 2008). The PEEK composites can be used for long-term load-bearing orthopedic, cardiovascular, and dental implant applications (Bakar et al. 2003). However, PEEK is bioinert and has limited ability to bind to natural bone tissue (Zheng et al. 2015). To overcome this issue, generally, hydroxyapatite (HA) is incorporated into PEEK, which has good biocompatibility and biodegradability, and together they can form strong bone bonding with bone tissue (Nga et al. 2015). In addition, HA also ameliorates the biological properties of PEEK-HA bionanocomposite scaffolds for bone tissue engineering applications, but the addition of HA into PEEK reduces the mechanical strength of scaffolds, especially in highly porous structures.

Converse and his co-workers fabricated polyetherketoneketone(PEKK) composite scaffolds with various porosities and volumes (0%, 20% and 40%) of HA whiskers as reinforcement, and suitable volumes of PEKK and HA (Converse et al. 2010). Sarasua and Remiro earlier fabricated short glass and carbon fiber (CF)-reinforced PEEK composites and reported on the improvement of mechanical properties (Sarasua and Remiro 1995). Their composites were fabricated by the injection molding method, and the maximum fiber reinforcement was 30% by weight. Unreinforced PEEK exhibits Young’s modulus of 3.1 GPa, whereas the introduction of 30% glass fibers and CFs increased Young’s modulus to 7.44 GPa and 12.38 GPa, respectively. Another research group fabricated a PEEK-CF acetabular cup, which was implanted in the body. After 28 months, they found that the wear depth in the retrieved insert was 0.130 mm, and the maximum wear was offset from the cup pole (Pace et al. 2008). Feng et al. fabricated a PEEK-HA nano-sandwich embedded with graphene nanosheets and carbon nanotubes (CMTs) to improve the mechanical properties for bone tissue engineering. CNTs prevent the agglomeration of the graphene nanosheets and increase the effective contact area between the reinforcement and the matrix. The loading ratio of the CNTs and graphene nanosheets was 8:2. The compressive strength and modulus of the scaffolds were increased by 63.58% and 56.54%, respectively, compared with those of the HAP/PEEK scaffolds (Feng et al. 2016). Wong et al. synthesized PEEK-strontium containing HA composites for load-bearing orthopedic applications (Wong et al. 2009). This composite was prepared using a compression molding technique, and the strontium-containing HA was 15–30 vol%. The bending modulus of the composites containing 25 and 30 vol% Sr-HA were 9.6 and 10.6 GPa and bending strength of 93.8 and 89.1 MPa, respectively. Besides, it was confirmed that HA-contained strontium enhanced the bioactivity in the PEEK composites. Scolozzi reported on PEEK for maxillary reconstruction. For more than 5 years, the patient was monitored and during this period, no complications were observed (Scolozzi 2012). Thus, PEEK is a biocompatible polymer that meets the functional requirement for implant materials is useful for bone scaffolding, and does not degrade at normal conditions (Shuai et al. 2016).

This study focuses on the fabrication, characterization, and mechanical properties of highly porous PEEK foam incorporated with nanomaterials. These bionanocomposites were fabricated with different percentages of porosity under an inert atmosphere to prevent oxidation of carbon at PEEK’s high melting temperature. Melt casting and salt porogen leaching methods were adopted for the fabrication of these highly porous bionanocomposite foams. HA, CFs and CNTs were incorporated to enhance the mechanical and biological properties of the scaffolds. The novelty of the present work is that, for the first time, highly robust PEEK scaffolds incorporated with various bone growth promoters (HA and carbon particles) were produced using the salt porogen technique, which are expected to improve the mechanical properties of the new bionanocomposites. The fundamental knowledge and skills gained through the study may be useful to advance the properties of functional scaffolds to address some of the problems in this field.

## Materials and methods

### Materials

The HA particles were purchased from Sigma–Aldrich at a size of less than 200 nm and a molecular weight of 502.31. HA is a prime mineral that forms the hard part of the bone (CaP), comprising by 50% of bone minerals. HA is a bioactive material that helps in bone growth and osteointegration in orthopedic applications. It has the following properties: reagent grade, synthetic powder, 1100 °C melting point, and insoluble in water. The polyacrylonitrile (PAN)-based carbon fiber with a diameter of 7 µm and length of 80–100 µm was provided by E&L Enterprises Inc. Catalytic multi-walled carbon nanotubes (MWCNTs) with a diameter of 140 nm and length of 7 µm were purchased from MER Corporation. Pure ocean salt (NaCl), ranging in size from 200 to 500 μm, was supplied by SaltWorks^®^ (Seattle, Washington). The high-performance, thermoplastic, unreinforced, and semi-crystalline PEEK 150P powder was obtained from Victrex. The tensile strength, tensile modulus, and elongation-at-break of PEEK are 100 MPa, 3.7 GPa, and 15%, respectively.

### Fabrication of PEEK bionanocomposite

#### Functionalization of CNTs/CF

Nanomaterials have high van der Waals forces due to their high surface area-to-volume ratio, which leads to their agglomeration, thus acting as an increaser of stress. The agglomeration of reinforcements, for instance, CNTs and CF in PEEK, is still a challenging issue due to the intrinsic inert nature of PEEK with organic solvents. The formation of strong covalent bonds among composite materials and uniform dispersion play a vital role in enhancing the mechanical properties of bionanocomposites. To obtain good covalent bonding between the CNTs/CF and PEEK matrix, chemical surface treatment is essential. Functionalization of the CNTs/CF with carboxylic-acid supports interfacial bonding and increases uniform dispersion. Nanoparticles and nitric acid were mixed in a ratio of 1:100 and stirred with a magnetic bar on a hot plate at an elevated temperature of 250 °C for 4 h at 500 rpm. Neutralization of this mixture was necessary to remove acid from the functionalized nanoparticles by mixing them with 750 mL of pure water, stirring them again for 10 min, and then applying a vacuum filter. This cycle was repeated at least 6 times to obtain effective neutralization. The neutralized and vacuumed-filtered CNTs/CF were dried in an oven overnight at a temperature of 85 °C.

#### Fabrication of PEEK bionanocomposites

PEEK is one of the leading semi-crystalline thermoplastic materials that emerged during the late 1990s as a replacement to metal implants primarily used for orthopedic and trauma applications. It is a hard-radiolucent plastic and has bone-like mechanical properties. The transparency characteristics of PEEK help doctors to accurately place the scaffold and easily monitor the healing process of the patient through imaging methods such as X-rays. It is stable at high temperatures (higher than 300 °C) and highly resistant to chemicals and radiation. As a matrix, PEEK is very compatible with many fibers or reinforcements. The required amounts of functionalized CNTs/CF (0.5, 1.0, and 2.0 wt%) with 20 mL of solvent (toluene) in a test tube were sonicated using a Sonics^®^ Vibra-Cell^™^ Model VCX 130 at 70% capacity for 15 min. Then, the required amount of PEEK and HA was added to the sonicated CNTs/CF, and 10 mL of solvent was further added to make a slurry for effective mixing. This mixture was sonicated in a 4 × 5-min cycle with a 5-min interval between each cycle. The solvent was drained from the sonicated slurry and allowed to dry for 48 h at room temperature and then further dried at 120 °C in an oven for 3 h. The dried mixture was hand-ground in a mortar for 2 min to break away any solid clusters. Then, salt porogen was added to the mixture and interspersed uniformly using a Fisher Scientific pulsing vortex mixer for 10 min at 3000 rpm to distribute it evenly and attain the proper pore size and properly interconnected pores.

**Fig. 1:**
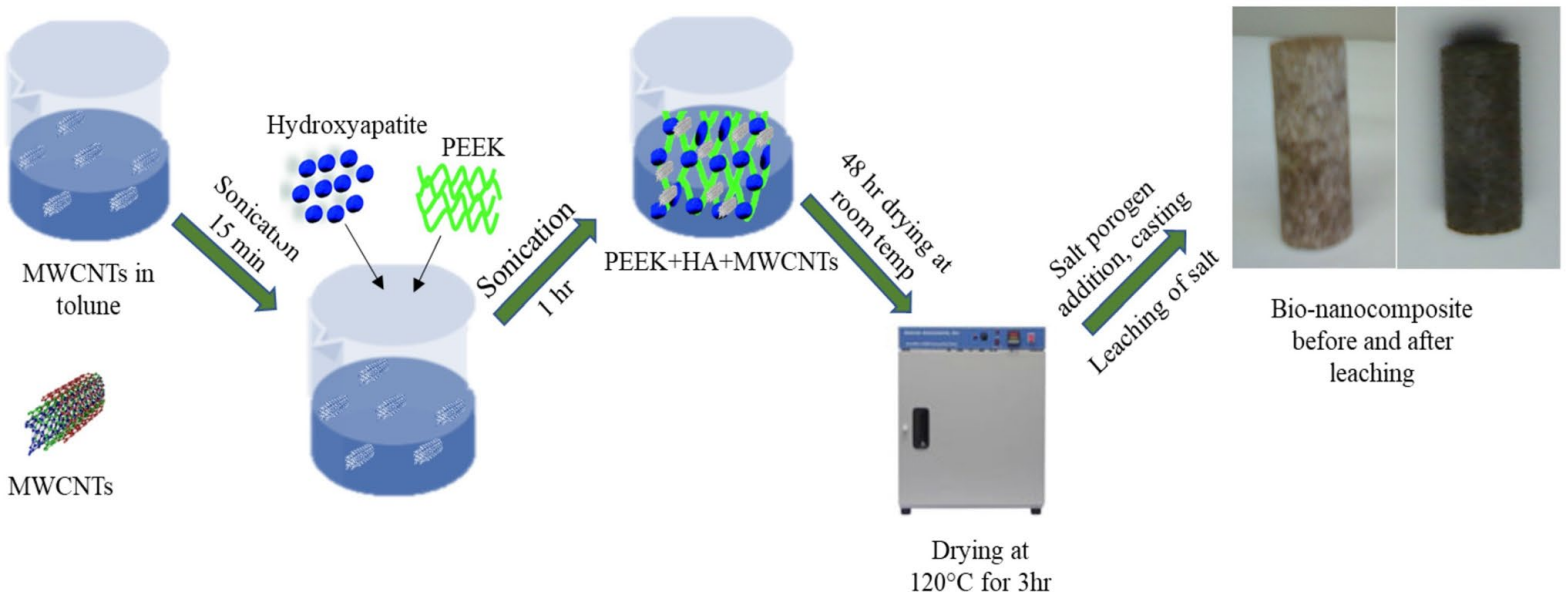
Schematic view of PEEK/HA/MWCNTs bionanocomposite synthesis process

Finally, the mixture was cast to obtain PEEK bionanocomposites. Casting involved two steps: (a) preparation of the mold, which facilitated proper releasing of the casted nanocomposite; and (b) melting of the nanocomposite mixture in an appropriate atmosphere without oxidization. The mold or die used was a 5/8-in aluminum tube to obtain a smooth circular-shaped casting to facilitate machining, and the ethanol-cleaned mold was sprayed with a high-temperature release agent (Slide, Hi-Temp 1800) and allowed to dry for 15 min. The nanocomposite/salt porogen mixture was placed in the mold, mixed lightly with a stiff wire, hand-pressed with an aluminum rod the same size as the mold, and wrapped with thin aluminum foil. Vent holes on the aluminum foil helped to release the gas produced during melting. The nanocomposite and salt porogen mixture was melted using a high-temperature Barnstead Thermodyne 1300 furnace in a regular atmosphere at 400 °C for 4 h and allowed to cool to room temperature within the furnace. The nanocomposite mixture was melted in an inert atmosphere using a Sentro Tech STT-1600-2.75-12 high-temperature vacuum tube furnace, to avoid oxidization from the presence of carbon, at 400 °C for 4 h at a pre-fixed ramp-up and cooling rate (4 °C per min). The filled molds were placed in the center of the furnace, and both ends of the furnace were sealed. Then, argon gas was pumped through the vacuum tube from a valve at one end and sealed. The casting was left to cool to room temperature within the furnace. Using a center lathe, the cast nanocomposite samples were machined to a size of 0.5 in diameter and 1 in length (as per ASTM D695, *L*/*D* = 2). A schematic of bionanocomposites fabrication process is presented in Fig. 1.

#### Leaching of salt porogen

The cast nanocomposite samples were machined to the required sizes (as per ASTM D695, *L*/*D* = 2) and then, leached using clean water for 3 days by changing the water at regular intervals of 6–8 h. During the water changing, samples were kept under a water flow for 10 min, which helped to remove any solid salt porogen particles. Then, the samples were vacuumed for 15 min in every 24 h of the leaching process. The samples were dried overnight at room temperature and, after that, further dried in an oven at 110 °C for 2 h to evaporate any trapped water droplets.

### Compression tests

Compression tests were carried out using different nanocomposite foams using an 810 Material Testing System (universal testing machine) according to ASTM-D1621, with a loading rate of 1.27 mm/min compression until the sample was compressed to 30% of its original size (original length 25.4 mm; after compression 7.62 mm). The compression modulus, yield strength, and strain-to-failure values were calculated from the tested data. It is noteworthy that the compression modulus of PEEK and its bionanocomposites was determined from the linear region of stress–strain curves. All experiments were repeated at least 5 times and the test results averaged.

### Micro-computed tomography test

Micro-computed tomography (CT) scanning makes it possible to compute and study the architecture and porosity of the PEKK scaffold. A porosity of 75% or higher is necessary to maintain spaces that are the right shape and size for tissue formation. Ceramic scaffolds for bones are required to have a pore size of 200–400 µm to enhance bone cell attachment; however, for liquid diffusion, pores of less than 10 µm are essential (Boyan et al. 1996). The ceramic content plays a major role in computing the foam morphology, mainly relative to porosity: the higher the content of ceramic particles, such as HA/CaP, the lower the porosity and specific surface. In one observation, the ceramic content reduced porosity from 81 to 74% without a ceramic filler. On the other hand, the addition of HA to PLA increased both wall thickness and pore size in the scaffolds. In cylindrical scaffolds, these two changes are typically due to the pore diameter gradient developed while the foam expands because cooling rates are different in the core and the outside surface. Other critical requirements for bone scaffolds are well-controlled interconnectivity of pores, which leads to proper cell attachment and preferred physical forms, thus providing support for vascularization of the in-growing tissue. For the best vascularization, a minimum of 100 µm pore size and porosity up to 90% are necessary (Karageorgiou and Kaplan 2005; Wei and Ma 2008). A composite scaffold in the as-prepared condition was examined for structural architecture and porosity using the ICT 80 Scanco medical micro-CT system. A section of a 2-mm scaffold was scanned, and two-dimensional images were generated, segmented, and reconstructed to create a three-dimensional binary image. The average porosity of the scaffold was measured by micro-CT unit, as explained in the following equation:

1 $$P_{\text{CT}} = \frac{{{\text{TV}} - {\text{MV}}}}{\text{TV}} \times 100 ,$$

where *P*_CT_ is the volume of porosity, TV is the total volume, and MV is the scanned material volume of the scaffold. Scanned data were analyzed to obtain the degree of anisotropy, pore size, and thickness of the connecting elements (Huang et al. 2008; Asmatulu et al. 2015; Uddin et al. 2019a, b). In this study, the porosity and architecture of the PEEK bionanocomposite were characterized using a Scanco Viva CT40 scanner (70 kV, 114 mA current) to determine pore size and interconnectivity. The scanning integration time was 200 ms. The bionanocomposite specimens of the various proportions of nanoparticles were scanned. However, only PEEK foam was scanned at different threshold values, because loose connectivity was observed with neat PEEK foams.

## Results and discussion

### Mechanical properties

**Fig. 2:**
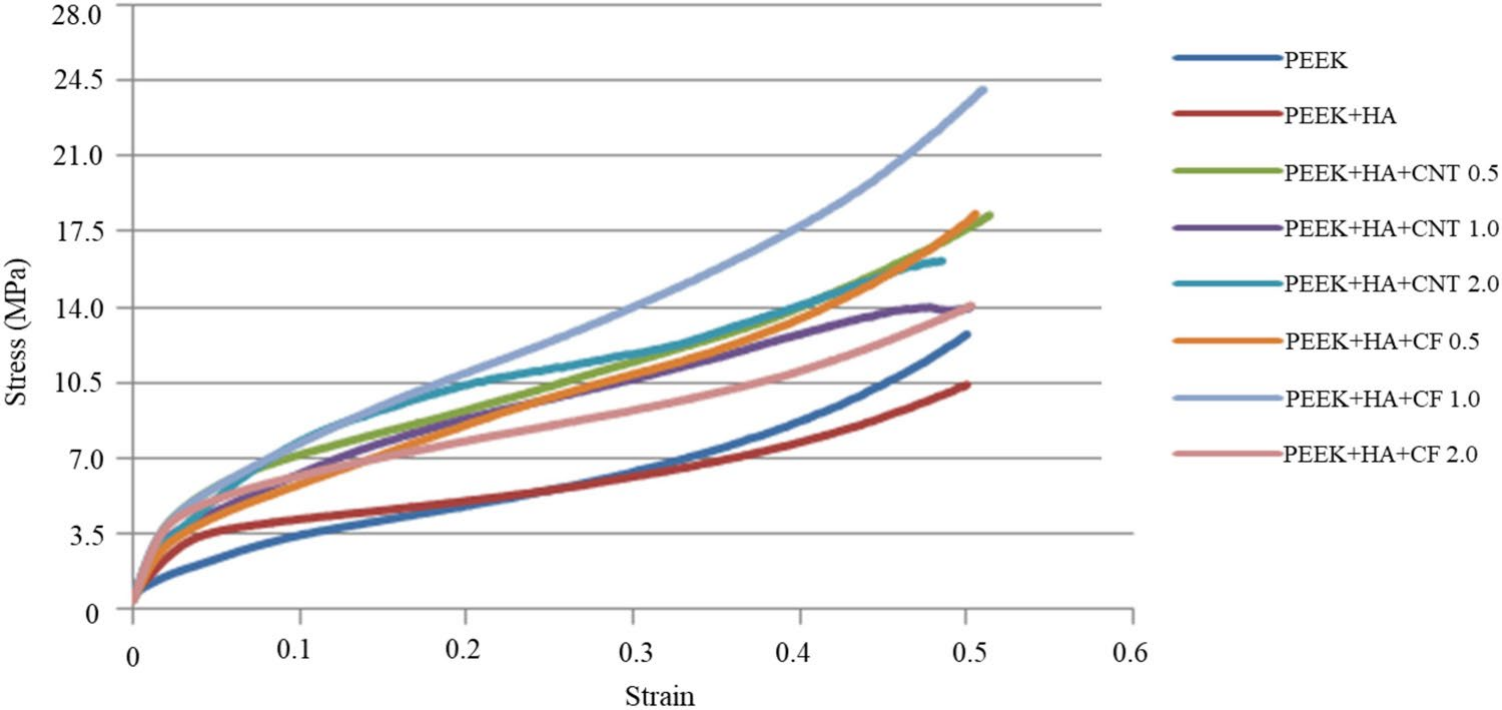
Typical compression stress–strain diagrams of neat PEEK, PEEK with 20 wt% HA and PEEK with 20 wt% HA plus 0.5, 1.0, and 2.0 wt% CNTs/CF bionanocomposite foams having 75% porosity

Bone strength can be defined as bone’s ability to withstand force (pulling or pushing), whereas flexibility is its ability to stretch without being deformed. Therefore, compressive/tensile strength is used to measure the strength of bone, and flexibility is measured by elasticity. In general, bones have a high compressive strength of 170 MPa and tensile strength of 104–121 MPa, but very low shear strength of 51.6 MPa (Turner et al. 2001). The most preferred failure mode is under tensile loading rather than compressive loading. Compression tests of the as-prepared bionanocomposite foams were performed for 75% and 85% porosities. The compression modulus of neat PEEK foam is about 66.46 MPa. However, the addition of 10–20 wt% HA into PEEK improved the modulus by almost 32%. In this study, 20 wt%, HA was chosen, since HA properties would enhance bone growth, and the chemical structure of HA is similar or the same as natural bone. Therefore, bionanocomposites containing PEEK with 20 wt% of HA and 0.5, 1.0, and 2.0 wt% CNTs/CF were fabricated, and their mechanical properties were measured. The representative stress–strain diagram of bionanocomposites as a function of nanomaterials loading for 75% porosities is presented in Fig. 2.

**Fig. 3:**
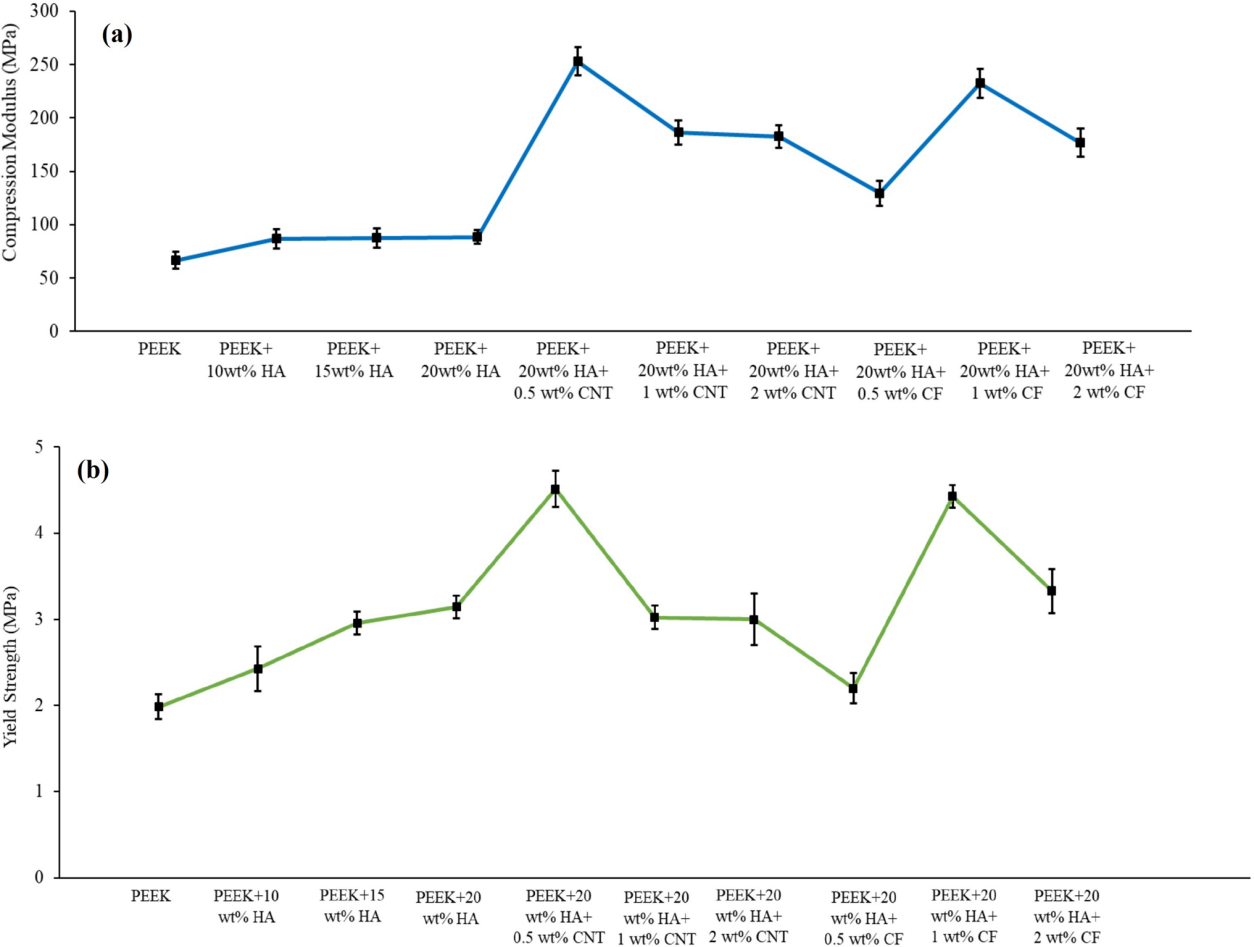
Bionanocomposites (75% porosity) containing CF and CNTs: **a** compression modulus and **b** yield strength

**Fig. 4:**
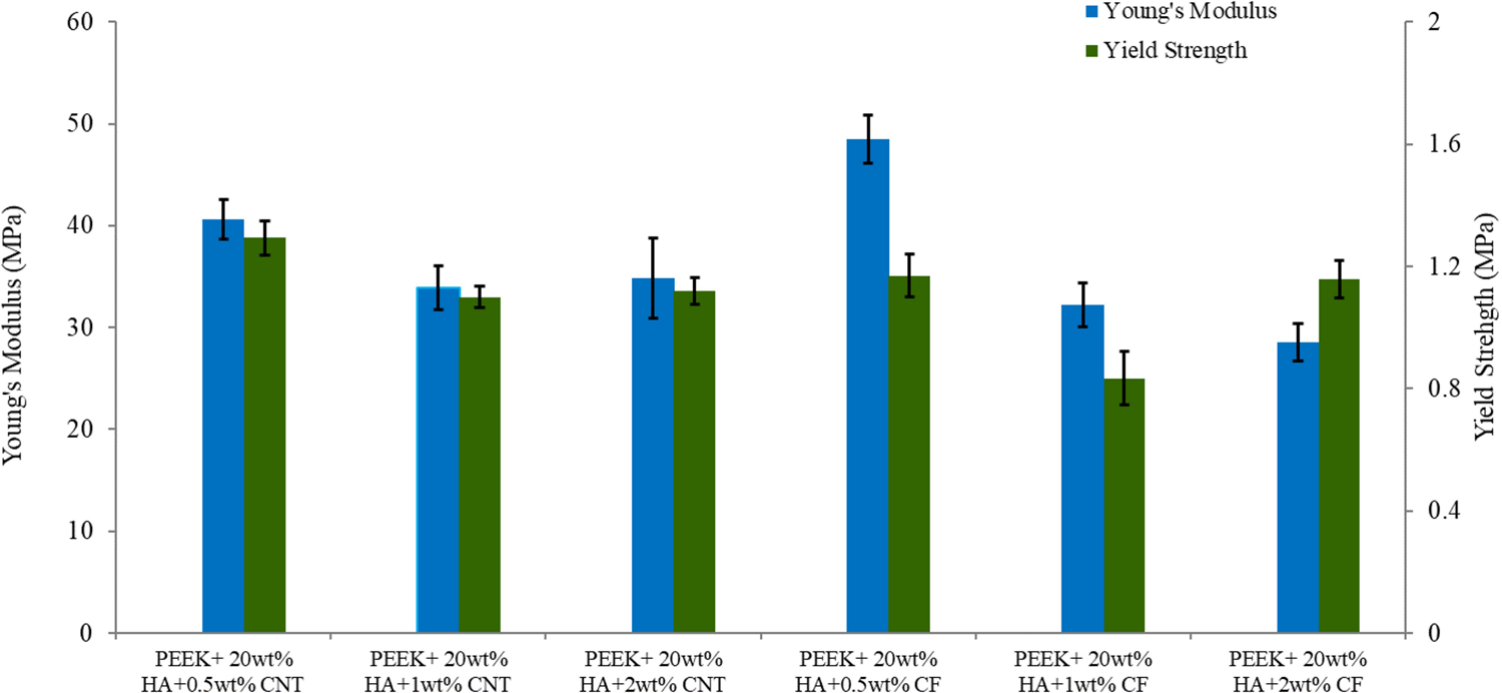
Bionanocomposites (85% porosity) containing CF and CNTs: modulus of elasticity and yield strength

At least five specimens were tested for each loading fraction and very small deviations in mechanical properties such as compression modulus, yield strength, and failure strain were observed that indicate the uniformity of the samples. Because of the 85% porosity, the neat PEEK foams were very soft and powdery, and thus a compression test could not be performed. The stress–strain plot of 75% and 85% porosity shows a similar trend. The compression modulus and yield strength of the bionanocomposites (75% porosity) containing CF and CNTs is illustrated in Fig. 3. The addition of CF and CNTs greatly enhanced the mechanical properties of the nanocomposites. The maximum results can be seen with the addition of 0.5 wt% CNTs and 1 wt% CF at 75% porosity. It is obvious that the compression modulus of PEEK/20 wt% HA can be increased from 88.32 to 252.91 MPa by adding only 0.5 wt% CNTs and to 232.45 MPa with the introduction of 1 wt% CF. About 186% and 163% enhancement of compression modulus was observed with the addition of 0.5 wt% CNTs and 1 wt% CF into the PEEK/20 wt% HA hybrid, respectively. However, when a higher amount of CNTs/CF was added, a down trend can be seen, which may be due to the agglomeration of excess CNTs/CF, which can function as a stressor concentration. However, uniformly dispersed CNTs into the PEEK matrix improved the mechanical properties, more so than the addition of CF. However, the PEEK/20 wt% HA/0.5 wt% CNT hybrid nanocomposite has a fracture elongation of 0.53%. Thus, the strain-at-break of the bionanocomposites slightly changes, as compared to the neat PEEK matrix studied here.The reduction of elongation-at-break of the bionanocomposites is not significant for all the nanocomposites studied here. Like the compression modulus, the 0.2% offset yield strength was derived from the stress–strain diagram, and it was observed that the bionanocomposites (75% porosity) containing 0.5 wt% CNTs and 1 wt% CF exhibit greater yield strength than the other nanocomposites studied here. The yield strength of PEEK/20 wt% HA can be increased from 3.14 to 4.51 MPa by adding only 0.5 wt% CNTs and 4.43 MPa with the introduction of 1 wt% CF. About 43% and 41% enhancement of yield strength was observed with the addition of 0.5 wt% CNTs and 1 wt% CF, respectively, into the PEEK/20 wt% HA hybrid. Figure 4 illustrates the compression modulus and yield strength of the bionanocomposites having 85% porosity. In this case, the bionanocomposites containing 2 wt% CNTs and 0.5 wt% CF presented higher modulus, and 0.5 wt% CNTs and 2 wt% CF presented greater 0.2% offset yield strength than the other nanocomposites. As recognized, CNTs and CF possess excellent stiffness and strength. Introduction of nanomaterials and inorganic HA into PEEK greatly enhances the mechanical properties as well as the biocompatibility of bionanocomposites for biomedical applications.

These results can be attributed to the uniform dispersion of functionalized CNTs and the formation of strong covalent bonds between PEEK molecules and HA nanoparticles. However, increasing the bionanocomposite porosity from 75 to 85% drastically reduced the mechanical properties.

The mechanical properties of cortical and cancellous bones have been reported by Mozafari et al. 2010. The cortical bone is denser than cancellous bone and more solid, with 5–30% porosity. However, cancellous bone has about 70–95% porosity (Keaveny et al. 2004). The cancellous bone has a strength of 0.1–30 MPa, elasticity of 0.02–0.5 GPA, and strain of 5–7% (Mozafari et al. 2010). The mechanical properties, i.e., compression modulus, yield strength, and elongation-at-break, of the bionanocomposite studied here fall within the range of cancellous bone.

**Fig. 5:**
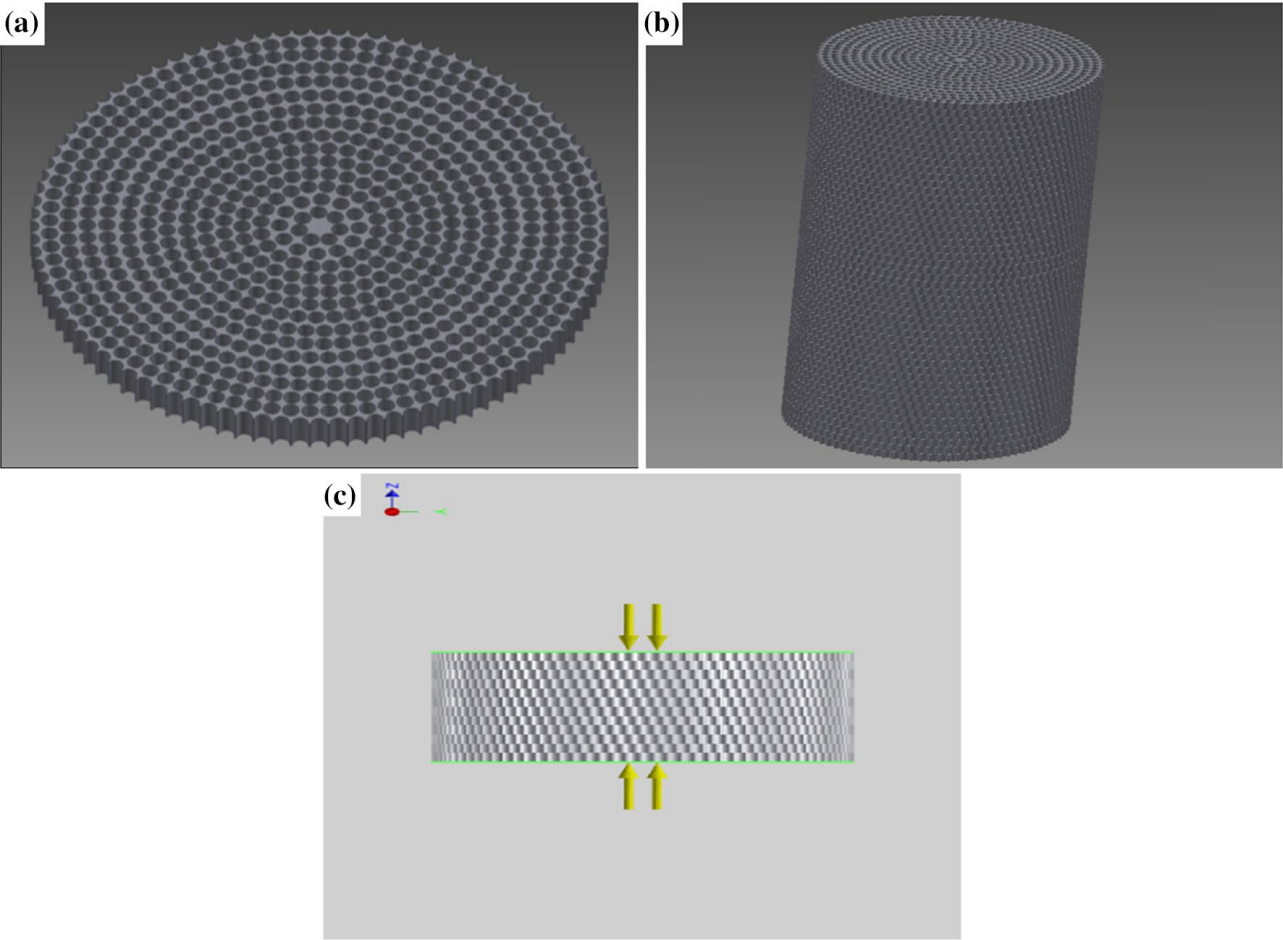
Foam model: **a** one layer, **b** stacked foam model to actual size, and **c** loading type

Analysis of the PEEK nanocomposites with 20 wt% HA and 0.5 wt% CNTs was performed on the model shown in Fig. 5a, b using AutoCAD Inventor 2013. About 12.7 mm diameter and tiny holes of 500 μm diameter, which are close to real pore size, were created to a thickness of 0.51 mm and stacked to a height of 25.4 mm to represent foam. Due to many holes, which results in a high volume of elements, computational modeling could not be performed for an actual height of 25.4 mm. Thus, a height of 6.35 mm was a reasonable height used to represent the foam model, and then the results were extended to 25.4 mm. Homogeneously distributed pressures of 1.38 MPa and 2.76 MPa were applied on both sides of the model faces, as shown in Fig. 5c, to generate a compression loading condition for simulation. The average Poison’s ratio of all materials was derived using the properties of the composite material. The actual density of the nanocomposite foam was calculated from the actual measured weight and volume. Simulation results of the mechanical properties of the bionanocomposite are comparable with the experimental results.

### Micro-CT (porosity) of bionanocomposites and structural analysis

**Fig. 6:**
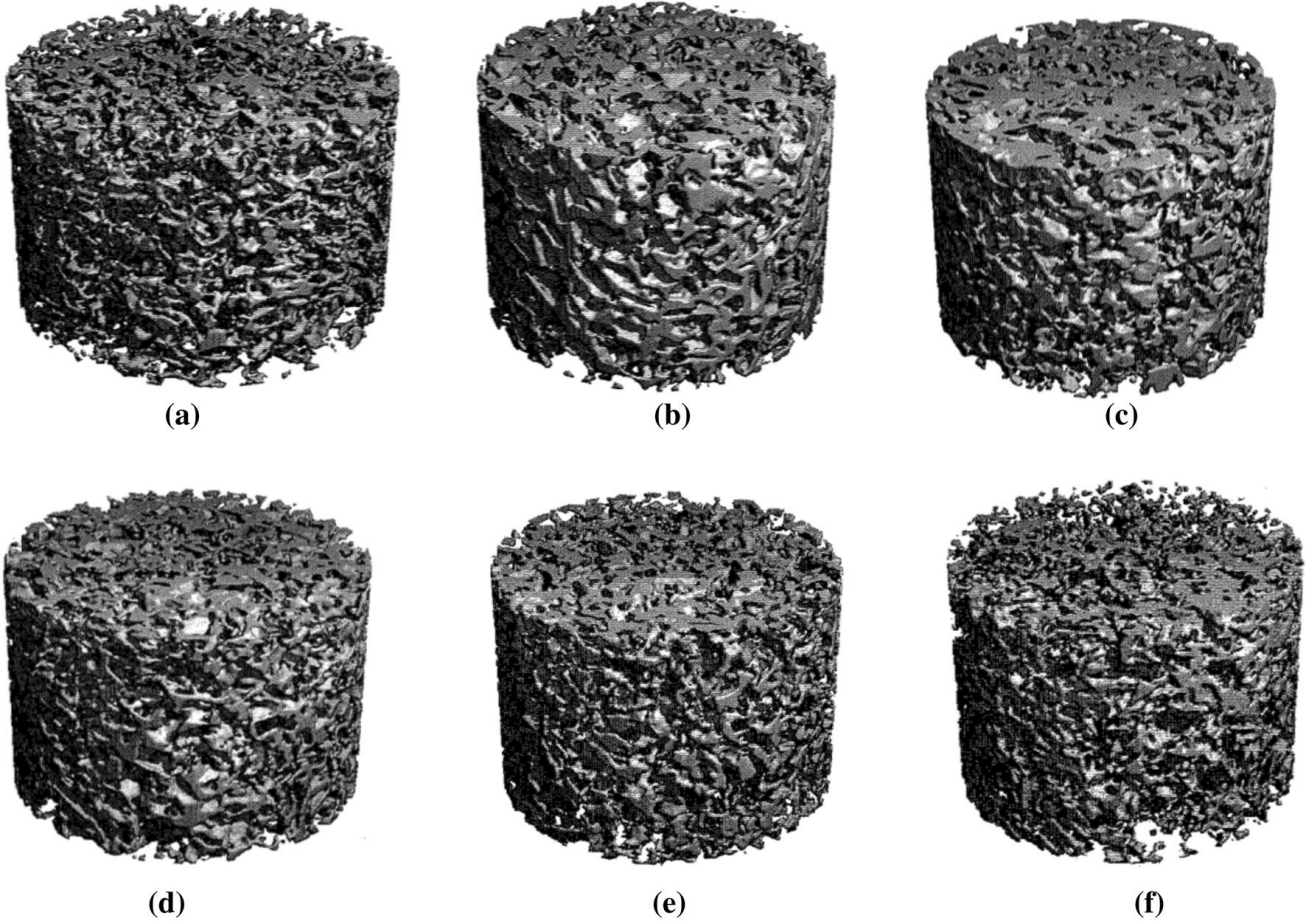
Micro-CT (porosity) images of bionanocomposites: (75% porosity): **a** neat PEEK, **b** PEEK with 20 wt% HA, **c** PEEK with 20 wt% HA and 0.5 wt% CNTs, and **d** PEEK with 20 wt% HA and 2.0 wt% CF; (85% porosity): **e** PEEK with 20 wt% HA and 0.5 wt% CNTs, **f** PEEK with 20 wt% HA and 2.0 wt% CF

The micro-computed tomography of the bionanocomposites having 75% and 85% porosity is summarized in Table 1. The average pore size values were received from the Micro-CT unit. The test was repeated five-six times to achieve reliable and repeatable test results. Pore size and interconnectivity of the nanocomposite foams are in order and within the designed sizes. In addition, the average pore size varies between 240 and 310 μm, which matches with optical microscopy size measurements because the size of the salt porogen used was between 200 and 500 μm. The degree of anisotropy confirms that salt porogen was mixed uniformly and effectively with 6–13% variation. However, neat PEEK with a higher percentage of porosity (85%) could not be tested due to the weak interconnectivity; in the case of 75% porosity, the variation of porosity is about 7% compared to the designed porosity. In addition, the nanocomposite with 2.0 wt% CNTs shows about 10% reduction, which may be due to the higher volume of CNTs. The micro-CT (porosity) images of bionanocomposites are illustrated in Fig. 6.

**Table 1:** Micro-CT (porosity) of bionanocomposites

Bionanocomposite	Design porosity (%)	Actual porosity (%)	Average pore size (µm)	Degree of anisotropy
PEEK + HA 20 wt% + CF 0.5 wt%	71.25	68.47	241 ± 3	1.0623
PEEK + HA 20 wt% + CF 2 wt%	81.75	80.72	309 ± 4	1.1074
PEEK + HA 20 wt% + CNT 0.5 wt%	71.25	61.57	251 ± 4	1.1152
PEEK + HA 20 wt% + CNT 2.0 wt%	81.75	77.3	292 ± 5	1.1059
PEEK + HA 20 wt%	71.25	68.8	275 ± 6	1.1146
Neat PEEK (75% porosity)	75	81.84	281 ± 4	1.1315
Neat PEEK (85% porosity)	N/A (due to weak interconnectivity)

As observed, the neat PEEK foam (75% porosity) appears as a spongy form compared to the PEEK with HA foams, as shown in Fig. 6a, b. Moreover, the addition of HA improved the interconnectivity of the PEEK foams. The nanocomposite foam (85% porosity) with CNTs had better interconnectivity compared to that with CF, which may be due to the higher volume of nanoparticles of CNTs compared to the microparticles of CF, as can be seen in Fig. 6e, f. In addition, the nanocomposite foam (85% porosity) with CF exhibits edges of loosely connected cells, as shown in Fig. 6f. However, the 75% porosity CNTs foams are considerably better connected than the 85% porosity CNTs foams. The nanoscale CNTs impart better connectivity than the microscale CF.

**Fig. 7:**
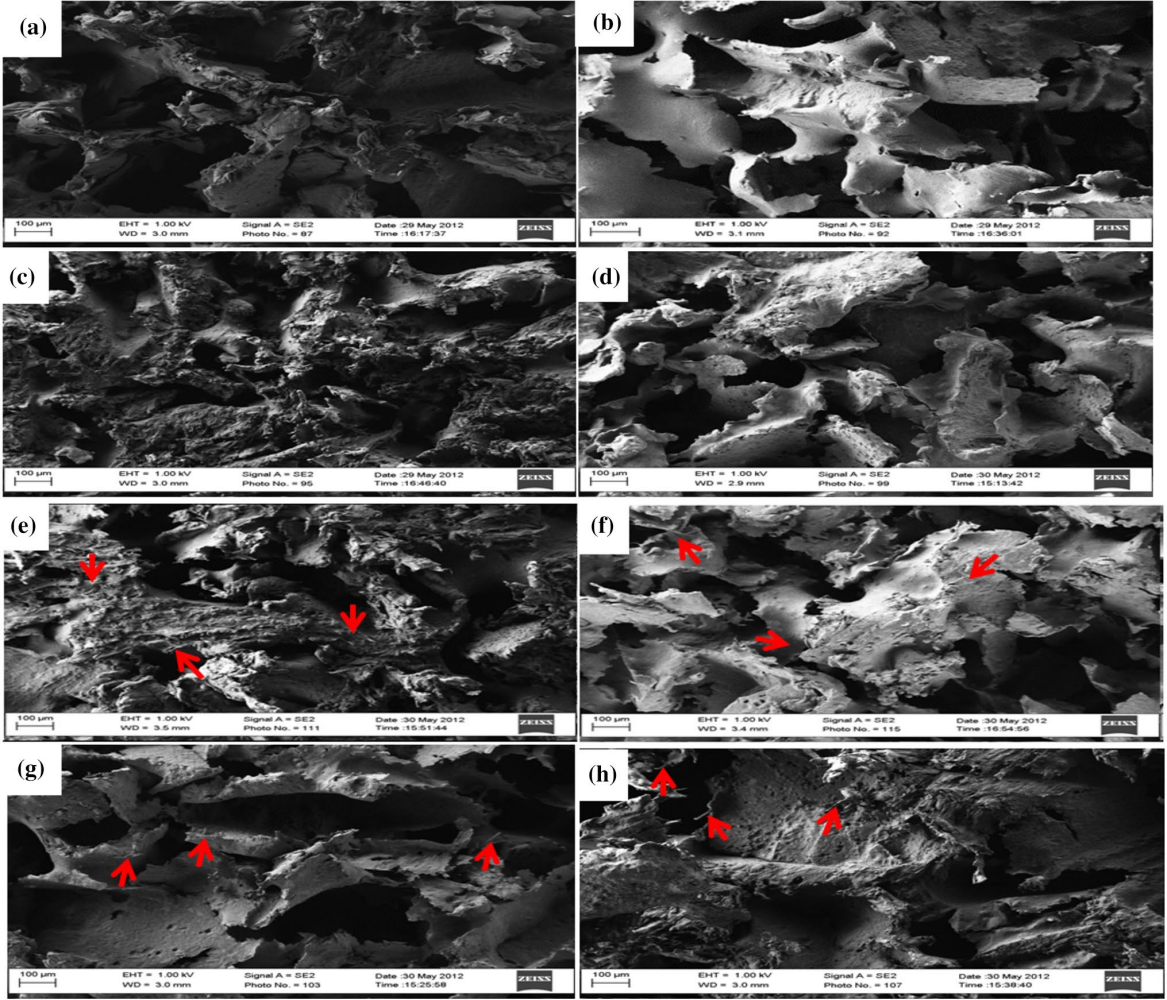
SEM images of bionanocomposites with 75% and 85% porosity (left and right columns, respectively): **a**, **b** neat PEEK; **c**, **d** PEEK with 20 wt% HA; **e**, **f** PEEK with 20 wt% HA and 0.5 wt% CNTs; **g**, **h** PEEK with 20 wt% HA and 1.0 wt% CF.n (Scale bar 100 µm, arrow sign indicates CNTs and CF)

Scanning electron microscopy (SEM) images of the bionanocomposites are shown in Fig. 7. In these SEM images, HA, CF and CNTs nanoparticles were embedded into PEEK polymer, so they were not clearly seen in those images. These images confirm the weak connectivity for higher porosities and greater concentrations for higher CNTs and CF reinforcement. However, the uniform size of porosity confirms that bionanocomposite foam can be fabricated with a uniform distribution of pores, pore size, and anisotropy in connectivity using cast melting and salt porogen leaching. The neat PEEK composite foam with 75% and 85% porosities are considerably dissimilar, as can be seen in Fig. 7a, 7b. The addition of HA into the neat PEEK shows a considerable strengthening of pore structures, as can be seen in Fig. 7c, d. Also, the incorporation of a higher amount of carbon particles (CF and CNTs) display enhanced concentration near the pore struts, as shown in Fig. 7e, f.

## Conclusions

In the present study, cast melting and salt porogen leaching fabrication techniques were used to fabricate PEEK foams with different HA, CF, and CNT nanoparticles, and porosity. This fabrication technique is greatly viable for highly porous foams, and 85% porosity could be achieved effectively. Compared with neat PEEK foam, the addition of HA, CF, and CNTs significantly enhanced the modulus and yield strength of the materials. The incorporation of 0.5 wt% CNTs to PEEK and HA resulted in greater mechanical properties than all other compositions studied in this work. As also observed in the compression tests, the compression modulus and yield strength of prepared bionanocomposites were 252.91 MPa and 4.51 MPa, as compared to neat PEEK 66.45 MPa and 1.98 MPa, respectively, for 75% porosity. In contrast, incorporating a higher amount of carbon particles into PEEK reduced the mechanical properties. This may be due to the agglomeration of the carbon particles, which act as stress increaser. In addition, the functionalization of CF and CNTs aided uniform dispersion through covalent bonding and resulted in higher mechanical strengths. The compression test could not be performed forthe higher 85% porosity neat PEEK foam because of the weak interconnectivity that was observed in the micro-CT test and by SEM image analysis. The micro-CT test further confirmed that the fabrication technique used in this study is reliable because foams had a uniform pore size and good interconnectivity, which will be useful for cell culturing and growth. Porosity variation compared to design porosity was ± 4%, except in the case of 85% porosity nanocomposite foam, which varied about 10%. The micro-CT tests also confirmed that the 85% porosity neat PEEK foam could not be scanned due to the loose interconnectivity. In biological tests (not reported here), nanocomposite foams proved to be non-toxic and had good cell viability, as shown in their mechanical performance, where a smaller addition of CNTs yielded better results. The above test results have proven that PEEK foams with HA, CF, and CNTs are a potential candidate for bone scaffold materials.
